# Factors associated with the use of complementary therapies in Taiwanese patients with systemic lupus erythematosus: a cross-sectional study

**DOI:** 10.1186/s12906-021-03416-w

**Published:** 2021-10-01

**Authors:** Ming-Chi Lu, Hui-Chin Lo, Hsiu-Hua Chang, Chia-Wen Hsu, Malcolm Koo

**Affiliations:** 1 Division of Allergy, Immunology and Rheumatology, Dalin Tzu Chi Hospital, Buddhist Tzu Chi Medical Foundation, Dalin, Chiayi Taiwan; 2 School of Medicine, Tzu Chi University, Hualien City, Taiwan; 3 Department of Medical Research, Dalin Tzu Chi Hospital, Buddhist Tzu Chi Medical Foundation, Dalin, Chiayi Taiwan; 4 Graduate Institute of Long-term Care, Tzu Chi University of Science and Technology, Hualien City, Hualien 970302 Taiwan; 5 Dalla Lana School of Public Health, University of Toronto, Toronto, ON Canada

**Keywords:** SLE, Complementary medicine, CAM, disease activity, Quality of life, Dietary supplements

## Abstract

**Background:**

This study aimed to investigate the prevalence of and the factors associated with the regular use of complementary therapies for Taiwanese patients with systemic lupus erythematosus (SLE).

**Methods:**

In this cross-sectional study, 351 patients with SLE were consecutively recruited from a regional hospital in southern Taiwan from April to August 2019. Demographic and clinical information, including the use of different types of complementary therapies, was ascertained using a self-constructed questionnaire. Disease-specific quality of life was measured using the Lupus Quality of Life (LupusQoL) questionnaire. SLE disease activity was assessed using the rheumatologist-scored Systemic Lupus Erythematosus Disease Activity Index 2000 (SLEDAI-2 K). Factors associated with the regular use of complementary therapies were evaluated using multiple logistic regression analyses.

**Results:**

Of the 351 patients with SLE, 90.3% were female, and 60.1% were ≥ 40 years of age. The prevalence of the regular use of any type of complementary therapy was 85.5%. The five most popular types of complementary therapy used were (1) fitness walking or strolling, (2) Buddhist prayer or attending temple, (3) vitamin consumption, (4) calcium supplementation, and (5) fish oil supplementation. Multiple logistic regression analyses revealed that the significant and independent factors associated with the regular use of complementary therapies in patients with SLE were age ≥ 40 years (adjusted odds ratio [aOR] 2.76, *p* = 0.013), nonoverweight or nonobesity (aOR 0.29, *p* = 0.004), engagement in vigorous exercise in the past year (aOR 4.62, *p* = 0.002), a lower SLEDAI-2 K score (aOR 0.90, *p* = 0.029), and a lower score in the physical health domain of the LupusQoL (aOR 0.57, *p* = 0.001).

**Conclusions:**

A high prevalence of complementary therapy use in Taiwanese patients with SLE was observed. Rheumatologists should routinely ask patients about their use of supplements to minimize the risk of interaction with medical therapy.

**Supplementary Information:**

The online version contains supplementary material available at 10.1186/s12906-021-03416-w.

## Background

Systemic lupus erythematosus (SLE) is a chronic autoimmune disease characterized by a diverse spectrum of clinical manifestations with a highly variable relapse-remission course. The disease predominantly affects women in reproductive years. Multiple organs or systems can be involved alone or in combination. Many patients can be affected by conditions such as malar rash, arthritis, oral ulcers, fatigue, and depression [1, 2]. A systematic review of epidemiological studies have indicated an increasing trend of SLE prevalence over time, with the highest estimates of prevalence in North America, at 241 per 100,000 people [3].

Despite recent advances in biological agents in addition to conventional treatments, such as nonsteroidal anti-inflammatory drugs, hydroxychloroquine, glucocorticoids, and immunosuppressive agents, mortality among SLE patients remains high. A study based on Taiwan’s National Health Insurance Research Database found that during the 2003–2008 study period, the mean prevalence and mortality rates were 97.5 and 1.2 per 100,000 people, respectively. Mortality within 1 year after diagnosis was 3.2% [4]. A meta-analysis of 15 reports including 26,101 patients with SLE revealed that the all-cause standardized mortality ratio was significantly increased in patients with SLE [5]. Furthermore, SLE is often associated with a number of comorbidities, such as cardiovascular disease, osteoporosis, and other autoimmune diseases. These comorbidities could adversely impact the health-related quality of life (QoL) of patients with SLE, which might in turn affect treatment adherence [6].

As there is no known cure for SLE, some patients may seek complementary therapies to control symptoms of their disease and the side effects of medications [7, 8]. Complementary therapies can be defined as “those therapies or modalities that are used adjunctively with biomedicine to augment healing, facilitate comfort, and promote health” [9]. A study of 707 patients with SLE in the United States, Canada, and the United Kingdom showed that approximately half of them used some form of complementary therapy in the past 6 months. In all three countries, relaxation techniques and massage were the two most commonly used therapies [10]. In another survey study of 192 Mexican patients with SLE, 53.6% of the respondents indicated that they were using or had used complementary therapies [11].

The reasons for using complementary therapies vary with the type of disease. In addition, a belief in a holistic notion of health and dissatisfaction with conventional medicine were associated with an increased use of complementary therapies [12]. Attitudes and beliefs, sex, disease-related factors, socioeconomic status, and cultural backgrounds were some of the contributing factors for complementary therapy use in patients with cancer [13]. However, few studies have explored the demographic and clinical factors associated with the use of complementary therapies in patients with SLE [11]. Therefore, this study aimed to investigate the prevalence of and the factors associated with the regular use of complementary therapies in Taiwanese patients with SLE.

## Methods

### Study design and population

A cross-sectional study design was used to consecutively recruit patients from the rheumatology outpatient clinic in a regional hospital in southern Taiwan between April and August 2019. The study was carried out in accordance with the Declaration of Helsinki. The study protocol approved by the institutional review board of Dalin Tzu Chi Hospital, Buddhist Tzu Chi Medical Foundation (No. B10801017). Written informed consent was obtained from all participants.

Based on an assumed prevalence of complementary therapy use of 70%, a power of 80% and a margin of error of 5%, the required sample size was estimated to be 318 [14]. To allow for a nonresponse of 10%, the sample size was increased to 350 individuals. The prevalence of 70% was estimated based on previous studies on complementary therapy use among healthy individuals [15] and those with chronic diseases in Taiwan [16].

### Inclusion and exclusion criteria

The inclusion criteria for this study were age ≥ 20 years and an SLE diagnosis based on the revised 1997 American College of Rheumatology (ACR) criteria [17] or the 2012 Systemic Lupus International Collaborating Clinics Classification Criteria (SLICC) [18]. Patients were excluded from the study if they had previously been diagnosed with any of the following major systemic autoimmune diseases: rheumatoid arthritis, systemic sclerosis, spondyloarthritis, polymyositis, dermatomyositis, or juvenile idiopathic arthritis.

### Measurement of demographic and clinical information

The patients were administered a paper-based questionnaire in Chinese that included closed-ended questions on age interval, sex, body mass index, educational level, marital status, job change due to SLE, employment status, self-perceived health status, disease duration of SLE, age at SLE diagnosis, alcohol use in the past year, smoking in the past year, vigorous exercise in the past year, and daily sleep duration. In addition, health-related QoL was ascertained by the disease-specific LupusQoL. The LupusQoL consists of 34 items across eight domains of QoL (physical health, emotional health, body image, pain, planning, fatigue, intimate relationships, and burden to others) [19]. Higher scores indicate a better health-related QoL. A Chinese version of the LupusQoL was used in this study. The scale was previously validated in 208 adult patients with SLE and was demonstrated to have construct validity comparable to similar domains of the EQ-5D. The test-retest reliability ranged from 0.84–0.97 [20]. The questionnaires were completed by the patients with assistance, if necessary, from two experienced research nurses of the rheumatology clinic.

Furthermore, the global disease activity of the SLE patients was scored with the Systemic Lupus Erythematosus Disease Activity Index 2000 (SLEDAI-2 K) by experienced rheumatologists. The scale was demonstrated to reflect disease activity at various levels comparable to the original SLEDAI  [21]. The SLEDAI-2 K is a global disease activity index composed of 24 descriptors reflecting nine organ systems. The score ranges from 0 to 105 points, with higher values indicating more severe disease activity.

### Measurement of complementary therapy use

There is currently no established way to categorize complementary therapies in research. Survey questions on the regular use of complementary therapies were adopted and modified from previous survey research studies conducted in Taiwan [22–24]. The questions in the questionnaire were broadly grouped in seven categories (Additional file 1), including (1) Body-based and energy therapy: Shiatsu or Tui Na (Chinese massage), chiropractic or osteopathic manipulation, Gua Sha therapy or cupping, acupuncture or moxibustion, and far-infrared therapy; (2) Mind-body therapy: qigong or Tai Chi, meditation or spiritual formation, relaxation therapy, and aromatherapy; (3) Folk remedies and religious practices: divination or nameology or fortune-changing, exorcism, Buddhist prayer or attending temple, and praying or attending church; (4) Exercise therapy: dancing, fitness workout, jogging, fitness walking, strolling, swimming, and cycling; (5) Chinese medicine: traditional Chinese medicine formulae and herbal remedies; (6) Nutrition supplements: vitamins, fish oil, ginkgo, calcium supplement, glucosamine, turmeric, probiotics, and (7) Diet therapy: raw food diet, organic diet, Mediterranean diet, low-carbohydrate diet, and ketogenic diet. Each of the categories also included an open-ended question for respondents to add other modalities of unlisted complementary therapies if necessary. A Likert-type response scale consisting of four choices (always use, occasionally use, have tried in the past, and never use) was used for all questions on the use of complementary therapies. These categories were recoded into two responses by treating only the “always use” category as “use”, with the remaining three categories collapsing into a “nonuse” category.

### Data analysis

Summary statistics are presented as frequencies with percentages or means with standard deviations (SD), as appropriate. Bivariate analyses comparing the use and nonuse of complementary therapies for demographic and clinical variables were conducted using the χ^2^ test or t-test. Moreover, univariate and multiple logistic regression analyses were conducted to determine factors associated with the use of complementary therapies for both overall (main outcome) and the top five most popular types (secondary outcomes) in patients with SLE. The top five most popular types of complementary therapies were selected based on their prevalence of use by the patients in this study.

In the univariate logistic regression analysis, variables with a *p* value of < 0.20 in their regression coefficients were entered and evaluated in the multiple logistic regression analysis. The backward likelihood ratio variable selection method was used to obtain the final model. All statistical analyses were conducted using IBM SPSS Statistics for Windows, version 25.0.0.2 (IBM Corp., Armonk, NY).

## Results

A total of 363 consecutively recruited and eligible patients were invited to join the study, and 351 (96.7%) agreed to participate. Table 1 shows the summary statistics for the demographic and clinical variables of the patients. Of the 351 patients, 90.3% were female, 60.1% were ≥ 40 years of age, 52.4% had a body mass index in the normal range, 73.8% indicated their own health as average or not healthy, 64.1% had SLE for ≥10 years, and 53.0% were diagnosed with SLE under the age of 30 years. The mean SLEDAI-2 K was 4.9 (SD 4.4). The mean scores for the eight domains of the LupusQoL were as follows: 81.3 (SD 19.9) for physical health, 83.2 (SD 19.8) for emotional health, 82.9 (SD 23.0) for body image, 80.2 (SD 26.5) for pain, 81.2 (SD 26.2) for planning, 72.0 (SD 23.5) for fatigue, 74.3 (SD 33.2) for intimate relationships, and 72.0 (SD 29.9) for burden to others.

**Table.1:** Univariate logistic regression analyses of factors associated with the use of complementary therapies among patients with systemic lupus erythematosus

Variable	n (%)			Odds ratio (95% CI)	P
	Total	Regular use of complementary therapies			
	351 (100)	Use 300 (85.5)	Non-use 51 (14.5)		
Sex					
Male	34 (9.7)	28 (82.4)	6 (17.6)	1	
Female	317 (90.3)	272 (85.8)	45 (14.2)	1.30 (0.51–3.30)	0.588
Age interval (years)					
20–39	140 (39.9)	110 (78.6)	30 (21.4)	1	
≥ 40	211 (60.1)	190 (90.0)	21 (10.0)	2.47 (1.35–4.52)	0.003
Body mass index					
Normal weight	184 (52.4)	165 (89.7)	19 (10.3)	1	
Underweight	48 (13.7)	41 (85.4)	7 (14.6)	0.67 (0.27–1.71)	0.407
Overweight or obesity	119 (33.9)	94 (79.0)	25 (21.0)	0.43 (0.23–0.83)	0.011
Educational level					
High school or below	177 (50.4)	150 (84.7)	27 (15.3)	1	
College or above	174 (49.6)	150 (86.2)	24 (13.8)	1.12 (0.62–2.04)	0.698
Marital status					
Single	118 (33.6)	98 (83.1)	20 (16.9)	1	
Married, widowed, or divorced	233 (66.4)	202 (86.7)	31 (13.3)	1.33 (0.72–2.45)	0.361
Job change related to SLE					
No	248 (70.7)	213 (85.9)	35 (14.1)	1	
Yes	103 (29.3)	87 (84.5)	16 (15.5)	0.89 (0.47–1.70)	0.731
Employment status					
Employed	222 (63.2)	186 (83.8)	36 (16.2)	1	
Unemployed	129 (36.8)	114 (88.4)	15 (11.6)	1.47 (0.77–2.81)	0.242
Self-report health status					
Healthy	92 (26.2)	78 (84.8)	14 (15.2)	1	
Not healthy or average	259 (73.8)	222 (85.7)	37 (14.3)	1.08 (0.55–2.10)	0.828
Disease duration, years					
< 10	126 (35.9)	104 (82.5)	22 (17.5)	1	
≥ 10	225 (64.1)	196 (87.1)	29 (12.9)	1.43 (0.78–2.61)	0.245
Age at diagnosis, years					
0–29	186 (53.0)	153 (82.3)	33 (17.7)	1	
≥ 30	165 (47.0)	147 (89.1)	18 (10.9)	1.76 (0.95–3.27)	0.072
Alcohol use in the past year					
Yes	81 (23.1)	61 (75.3)	20 (24.7)	1	
No	270 (76.9)	239 (88.5)	31 (11.5)	2.53 (1.35–4.74)	0.004
Smoking in the past year					
Yes	34 (9.7)	25 (73.5)	9 (26.5)	1	
No	317 (90.3)	275 (86.8)	42 (13.2)	2.36 (1.03–5.40)	0.042
Vigorous exercise in the past year					
Never	60 (17.1)	43 (71.7)	17 (28.3)	1	
Yes	291 (82.9)	257 (88.3)	34 (11.7)	2.99 (1.54–5.82)	0.001
Sleep duration/day, hours					
≥ 8	69 (19.7)	43 (71.7)	17 (28.3)	1	
≤ 7	282 (80.3)	257 (88.3)	34 (11.7)	2.99 (1.54–5.82)	0.001
Sleeping medication use					
No	253 (72.1)	210 (83.0)	43 (17.0)	1	
Yes	98 (27.9)	90 (91.8)	8 (8.2)	2.30 (1.04–5.10)	0.039
SLEDAI-2 K, mean (SD) (*n* = 333)	4.9 (4.4)	4.7 (4.1)	6.2 (5.9)	0.94 (0.88–1.00)	0.035
Domain of LupusQoL, mean (SD)					
Physical health	81.3 (19.9)	80.1 (20.0)	88.4 (17.6)	0.74 (0.59–0.92)	0.008
Emotional health	83.2 (19.8)	82.8 (19.9)	85.5 (18.8)	0.92 (0.78–1.09)	0.362
Body image (*n* = 340)	82.9 (23.0)	83.0 (22.8)	82.5 (24.8)	1.01 (0.89–1.15)	0.904
Pain	80.2 (26.5)	79.7 (26.9)	83.5 (23.6)	0.94 (0.83–1.07)	0.344
Planning	81.2 (26.2)	80.7 (26.7)	84.6 (23.0)	0.94 (0.83–1.06)	0.319
Fatigue	72.0 (23.5)	71.4 (23.7)	75.5 (21.9)	0.92 (0.81–1.06)	0.253
Intimate relationships (*n* = 271)	74.3 (33.2)	72.9 (34.6)	81.4 (23.7)	0.92 (0.82–1.03)	0.129
Burden to others	72.0 (29.9)	71.4 (30.3)	79.1 (27.4)	0.91 (0.81–1.02)	0.094

*CI* confidence interval, *SD* standard deviation, *SLE* systemic lupus erythematosus, *SLEDAI-2 K* Systemic Lupus Erythematosus Disease Activity Index 2000

The score of LupusQoL was multiplied by 10 in the regression analysis, and therefore, the odds ratio was per 10-point change in LupusQoL

The results of the univariate logistic regression analyses on the use of complementary therapies for each of the demographic and clinical variables are also shown in Table 1. The prevalence of the use of any type of complementary therapy was 85.5%. An increased use of complementary therapies was found to be significantly associated with the following variables: ≥ 40 years of age (odds ratio [OR] 2.47, *p* = 0.003), no alcohol use in the past year (OR 2.53, *p* = 0.004), not smoking in the past year (OR 2.36, *p* = 0.042), engagement in vigorous exercise in the past year (OR 2.99, *p* = 0.001), ≤ 7 h of sleep per day (OR 2.99, *p* = 0.001), use of sleeping medication (OR 2.30, *p* = 0.039), a lower SLEDAI-2 K score (OR 0.94, *p* = 0.035), and a lower score in the physical health domain of the LupusQoL (OR 0.74, *p* = 0.008). Conversely, a decreased use of complementary therapies was significantly associated with overweight or obesity (OR 0.43, *p* = 0.011).

**Fig. 1:**
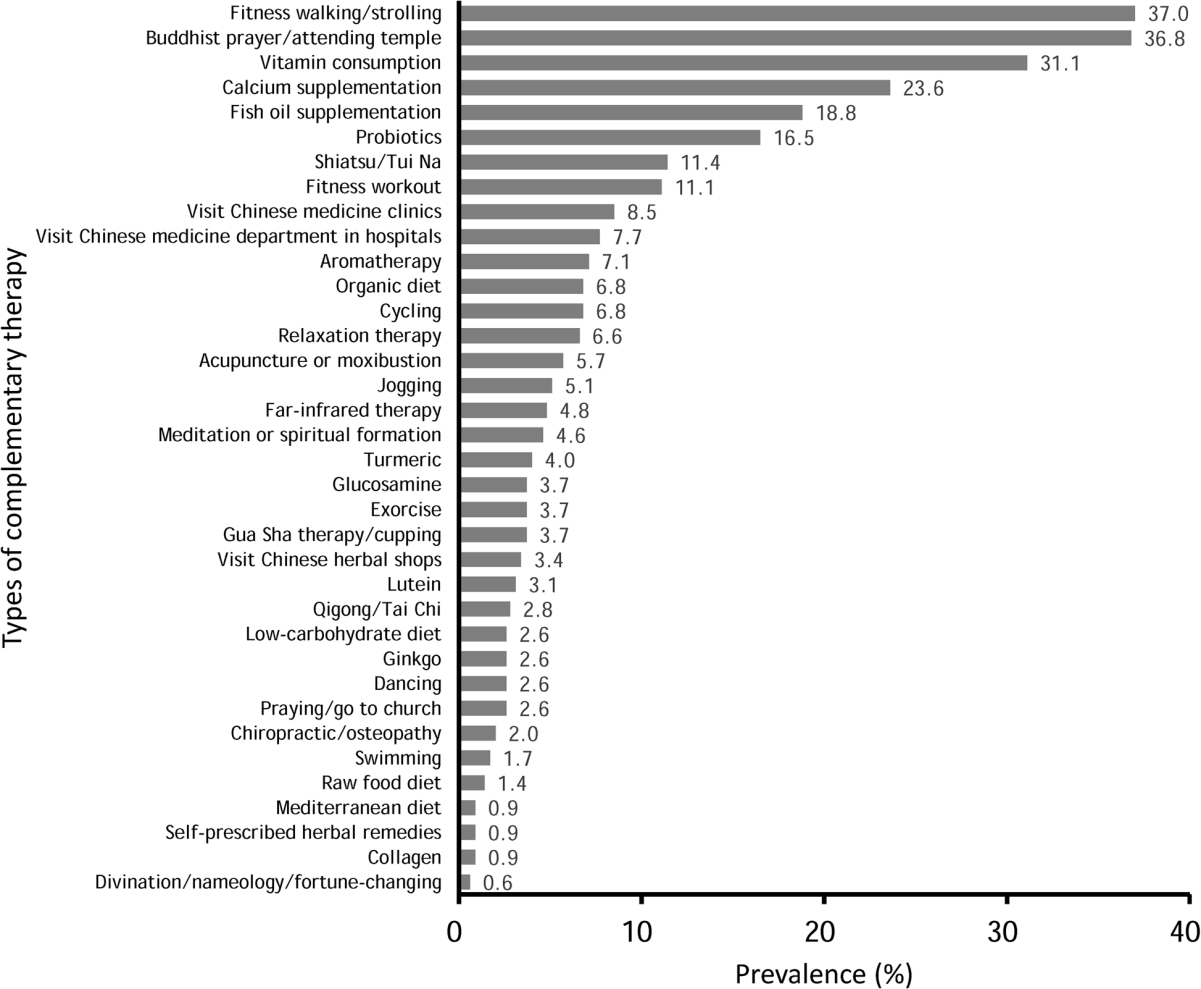
Prevalence of the use of different complementary therapies in patients with systemic lupus erythematosus

Based on the prevalence of complementary therapy use in the patients of this study, the top five types of complementary therapies used by the study participants were fitness walking or strolling (37.0%), Buddhist prayer or attending temple (36.8%), vitamin consumption (31.1%), calcium supplementation (23.6%), and fish oil supplementation (18.8%) (Fig. 1). The results of the univariate logistic regression analyses for these five types of complementary therapies are shown in Table 2. First, an increased use of fitness walking or strolling was found to be significantly associated with age ≥ 40 years (OR 2.46, *p* < 0.001), a marital status of married, widowed, or divorced (OR 1.63, *p* = 0.043), unemployment (OR 2.21, *p* = 0.001), a disease duration of ≥10 years (OR 1.70, *p* = 0.027), SLE diagnosis at ≥30 years of age (OR 2.20, *p* < 0.001), no smoking in the past year (OR 4.95, *p* = 0.003), engagement in vigorous exercise in the past year (OR 10.69, *p* < 0.001), sleep duration of ≤7 h per day (OR 1.87, *p* = 0.037), and a lower SLEDAI-2 K score (OR 0.92, *p* = 0.003).

**Table.2:** Univariate logistic regression analyses of factors associated with the use of the top five most popular complementary therapies among patients with systemic lupus erythematosus

Variable	Fitness walking/strolling			Buddhist prayer/attending temple			Vitamin consumption			Calcium supplementation			Fish oil supplementation		
	% use	Odds ratio	*p*	% use	Odds ratio	*p*	% use	Odds ratio	*p*	% use	Odds ratio	*p*	% use	Odds ratio	*p*
Sex															
Male	32.4	1		32.4	1		23.5	1		14.7	1		11.8	1	
Female	37.5	1.26	0.552	37.2	1.24	0.576	31.9	1.52	0.321	24.6	1.89	0.203	19.6	1.82	0.275
Age interval (years)															
20–39	25.0	1		25.0	1		25.7	1		13.6	1		19.3	1	
≥ 40	45.0	2.46	< 0.001	44.5	2.41	< 0.001	34.6	1.53	0.079	30.3	2.77	< 0.001	18.5	0.95	0.851
Body mass index															
Normal weight	37.0	1		34.8	1		31.5	1		23.4	1		22.8	1	
Underweight	37.5	1.02	0.945	41.7	1.34	0.378	35.4	1.19	0.608	29.2	1.35	0.407	16.7	0.68	0.358
Overweight or obesity	37.0	1.00	0.997	37.8	1.14	0.591	28.6	0.87	0.586	21.8	0.92	0.758	13.4	0.52	0.045
Educational level															
High school or below	40.1	1		42.9	1		27.7	1		26.6	1		14.1	1	
College or above	33.9	0.77	0.229	30.5	0.58	0.016	34.5	1.38	0.169	20.7	0.72	0.197	23.6	1.87	0.025
Marital status															
Single	29.7	1		28.8	1		30.5	1		16.1	1		16.9	1	
Married, widowed, or divorced	40.8	1.63	0.043	40.8	1.70	0.029	31.3	1.04	0.875	27.5	1.97	0.019	19.7	1.20	0.527
Job change related to SLE															
No	37.1	1		38.3	1		29.4	1		21.8	1		20.2	1	
Yes	36.9	0.99	0.971	33.0	0.79	0.349	35.0	1.29	0.310	28.2	1.41	0.201	15.5	0.73	0.314
Employment status															
Employed	30.2	1		35.6	1		30.2	1		19.4	1		21.6	1	
Unemployed	48.8	2.21	0.001	38.8	1.15	0.552	32.6	1.12	0.643	31.0	1.87	0.014	14.0	0.59	0.078
Self-report health status															
Healthy	34.8	1		32.6	1		22.8	1		16.3	1		26.1	1	
Not healthy or average	37.8	1.14	0.602	38.2	1.28	0.338	34.0	1.74	0.049	26.3	1.83	0.056	16.2	0.55	0.039
Disease duration, years															
< 10	29.4	1		30.2	1		22.2	1		16.7	1		16.7	1	
≥ 10	41.3	1.70	0.027	40.4	1.57	0.056	36.0	1.97	0.008	27.6	1.90	0.023	20.0	1.25	0.444
Age at diagnosis, years															
0–29	28.5	1		30.6	1		32.3	1		21.5	1		22.6	1	
≥ 30	46.7	2.20	< 0.001	43.6	1.75	0.012	29.7	0.89	0.605	26.1	1.29	0.317	14.5	0.58	0.056
Alcohol use															
Yes	29.6	1		21.0	1		29.6	1		17.3	1		23.5	1	
No	39.3	1.54	0.117	41.5	2.67	0.001	31.5	1.09	0.752	25.6	1.64	0.127	17.4	0.69	0.223
Smoking															
Yes	11.8	1		23.5	1		20.6	1		8.8	1		8.8	1	
No	39.7	4.95	0.003	38.2	2.01	0.098	32.2	1.83	0.170	25.2	3.49	0.043	19.9	2.56	0.130
Vigorous exercise in the past year															
Never	6.7	1		21.7	1		31.7	1		13.3	1		10.0	1	
Yes	43.3	10.69	< 0.001	39.9	2.40	0.009	30.9	0.97	0.910	25.8	2.26	0.043	20.6	2.34	0.061
Sleep duration/day, hours															
≥ 8	26.1	1		29.0	1		36.2	1		23.2	1		16.7	1	
≤ 7	39.7	1.87	0.037	38.7	1.54	0.137	29.8	0.75	0.301	23.8	1.03	0.920	27.5	0.53	0.040
Sleeping medication use															
No	37.2	1		35.6	1		27.3	1		22.5	1		18.6	1	
Yes	36.7	0.98	0.942	39.8	1.20	0.462	40.8	1.84	0.015	26.5	1.24	0.429	19.4	1.05	0.862
SLEDAI-2 K		0.92	0.003		0.98	0.529		0.96	0.125		1.01	0.789		1.02	0.479
Domain of LupusQoL															
Physical health		0.90	0.052		0.87	0.011		0.89	0.045		0.85	0.005		1.14	0.108
Emotional health		1.04	0.498		0.97	0.641		1.01	0.895		0.94	0.336		0.99	0.841
Body image		1.04	0.474		0.97	0.472		0.98	0.756		0.99	0.890		1.01	0.885
Pain		1.00	0.921		0.92	0.056		0.99	0.895		0.94	0.132		1.07	0.257
Planning		1.02	0.709		0.94	0.175		0.97	0.448		0.92	0.062		1.08	0.217
Fatigue		1.06	0.194		0.96	0.342		0.93	0.134		0.98	0.706		0.99	0.815
Intimate relationships		0.96	0.220		0.89	0.002		0.99	0.753		0.87	< 0.001		1.03	0.560
Burden to others		1.00	0.987		0.90	0.006		0.98	0.506		0.98	0.543		0.97	0.437

*SLE* systemic lupus erythematosus, *SLEDAI-2 K* Systemic Lupus Erythematosus Disease Activity Index 2000

The score of LupusQoL was multiplied by 10 in the regression analysis, and therefore, the odds ratio was per 10-point change in LupusQoL

Second, increased Buddhist praying or attendance to temple was found to be significantly associated with ≥40 years of age (OR 2.41, *p* < 0.001), marital status of being married, widowed, or divorced (OR 1.70, *p* = 0.029), SLE diagnosis at ≥30 years of age (OR 1.75, *p* = 0.012), not using alcohol in the past year (OR 2.67, *p* = 0.001), engaged in vigorous exercise in the past year (OR 2.40, *p* = 0.009), and a lower score in three domains of the LupusQoL: physical health domain (OR 0.87, *p* = 0.011), intimate relationships (OR 0.89, *p* = 0.002), and burden to others (OR 0.90, *p* = 0.006). Conversely, decreased Buddhist praying or attendance to temple was significantly associated with an educational level of college or above (OR 0.58, *p* = 0.016).

Third, an increased use of vitamins was found to be significantly associated with a self-reported health status of not healthy or average (OR 1.74, *p* = 0.049), a disease duration of ≥10 years (OR 1.97, *p* = 0.008), sleeping medication use (OR 1.84, *p* = 0.015), and a lower score in the physical health domain of the LupusQoL (OR 0.89, *p* = 0.045).

Fourth, an increased use of calcium supplements was found to be significantly associated with ≥40 years of age (OR 2.77, *p* < 0.001), marital status of married, widowed, or divorced (OR 1.97, *p* = 0.019), unemployment (OR 1.87, *p* = 0.014), a disease duration of ≥10 years (OR 1.90, *p* = 0.023), not smoking in the past year (OR 3.49, *p* = 0.043), engagement in vigorous exercise in the past year (OR 2.26, *p* = 0.043), and a lower score in two domains of the LupusQoL: physical health (OR 0.85, *p* = 0.005) and intimate relationships (OR 0.87, *p* < 0.001).

Fifth, an increased use of fish oil supplements was found to be significantly associated with an educational level of college or above (OR 1.87, *p* = 0.025). Conversely, a decreased use of fish oil supplements was significantly associated with overweight or obesity (OR 0.52, *p* = 0.045), a self-reported health status of not healthy or average (OR 0.55, *p* = 0.039), and sleep duration of ≤7 h per day (OR 0.53, *p* = 0.040).

In addition, the results of multiple logistic regression analyses of the primary and secondary outcomes are summarized in Table 3. The significant and independent factors associated with the regular use of complementary therapies in patients with SLE were ≥ 40 years of age (adjusted OR 2.76, *p* = 0.013), nonoverweight or nonobesity (adjusted OR 0.29, *p* = 0.004), engagement in vigorous exercise in the past year (adjusted OR 4.62, *p* = 0.002), a lower SLEDAI-2 K score (adjusted OR 0.90, *p* < 0.029), and a lower score in the physical health domain of the LupusQoL (adjusted OR 0.57, *p* = 0.001).

**Table.3:** Multiple logistic regression analyses of factors associated with the use of complementary therapies and the top five most popular complementary therapies among patients with systemic lupus erythematosus

Variable	Regular use of complementary therapies		Fitness walking/strolling		Buddhist prayer/attending temple		Vitamin consumption		Calcium supplementation		Fish oil supplementation	
	AOR (95% CI)	*p*	AOR (95% CI)	*p*	AOR (95% CI)	*p*	AOR (95% CI)	*p*	AOR (95% CI)	*p*	AOR (95% CI)	*p*
Age interval (years)												
20–39	1				1		1					
≥ 40	2.76 (1.24–6.15)	0.013			3.47 (1.90–6.34)	< 0.001	2.14 (1.22–3.77)	0.008				
Body mass index												
Normal weight	1											
Underweight	1.15 (0.36–3.64)	0.810										
Overweight or obesity	0.29 (0.13–0.70)	0.004										
Educational level												
High school or below							1				1	
College and above							2.63 (1.51–4.57)	0.001			1.93 (1.11–3.35)	0.020
Employment status												
Employed			1									
Unemployed			2.22 (1.26–3.93)	0.006								
Self-report health status												
Healthy							1					
Not healthy or average							2.01 (1.12–3.62)	0.020				
Disease duration, years												
< 10 years							1					
≥ 10 years							2.04 (1.20–3.49)	0.009				
Alcohol use												
Yes					1							
No					2.39 (1.16–4.92)	0.018						
Smoking												
Yes			1									
No			3.98 (1.22–12.94)	0.022								
Vigorous exercise in the past year												
Never	1		1		1				1			
Yes	4.62 (1.75–12.18)	0.002	16.74 (5.16–54.29)	< 0.001	2.49 (1.09–5.70)	0.031			3.49 (1.16–10.54)	0.027		
Sleep duration/day, hours												
≥ 8											1	
≤ 7											0.51 (0.27–0.94)	0.032
SLEDAI-2 K	0.90 (0.83–0.99)	0.029	0.92 (0.86–0.98)	0.014								
Domain of LupusQoL mean (SD)												
Physical health	0.57 (0.41–0.80)	0.001	0.76 (0.64–0.91)	0.003								
Fatigue			1.15 (1.01–1.31)	0.034								
Intimate relationships									0.86 (0.79–0.93)	< 0.001		
Burden to others					0.87 (0.79–0.96)	0.004						

*AOR* adjusted odds ratio, *SLEDAI-2 K* Systemic Lupus Erythematosus Disease Activity Index 2000

The score of LupusQoL was multiplied by 10 in the regression analysis, and therefore, the odds ratio was per 10-point change in LupusQoL

Table 3 also shows the factors associated with the five secondary outcomes. First, for the use of fitness walking or strolling, the significant and independent factors included employment (adjusted OR 2.22, *p* = 0.006), not smoking in the past year (adjusted OR 3.98, *p* = 0.022), engagement in vigorous exercise in the past year (adjusted OR 16.74, *p* < 0.001), a lower SLEDAI-2 K score (adjusted OR 0.92, *p* = 0.014), a lower score in the physical health (adjusted OR 0.76, *p* = 0.003) domain of the LupusQoL, and a higher score in the fatigue (adjusted OR 1.15, *p* = 0.034) domain of the LupusQoL.

Second, for Buddhist prayer or attendance to temple, the significant and independent factors included age ≥ 40 years (adjusted OR 3.47, *p* < 0.001), no alcohol use in the past year (adjusted OR 2.39, *p* = 0.018), engagement in vigorous exercise in the past year (adjusted OR 2.49, *p* = 0.031), and a lower score in the burden to others domain of the LupusQoL (adjusted OR 0.87, *p* = 0.004).

Third, for the use of vitamins, the significant and independent factors included ≥40 years of age (adjusted OR 2.14, *p* = 0.008), educational level of college or above (adjusted OR 2.63, *p* = 0.001), a self-reported health status of not healthy or average (adjusted OR 2.01, *p* = 0.020), and a disease duration of ≥10 years (adjusted OR 2.04, *p* = 0.009).

Fourth, for the use of calcium supplementation, the significant and independent factors included engagement in vigorous exercise in the past year (adjusted OR 3.49, *p* = 0.027) and a lower score in the intimate relationships domain of the LupusQoL (adjusted OR 0.86, *p* < 0.001).

Fifth, for the use of fish oil supplements, the significant and independent factors included an educational level of college or above (adjusted OR 1.93, *p* = 0.020) and sleep duration of ≤7 h per day (adjusted OR 0.51, *p* = 0.032).

## Discussion

This cross-sectional study investigated the prevalence of and factors associated with the regular use of complementary therapies in patients with SLE. The prevalence of the use of any type of complementary therapy was 85.5%, which is similar to the 82.4% reported in a nationwide survey study on patients with breast cancer in Taiwan [23] and the 86.9% reported in a population-based survey among 2310 Taiwanese adults [25]. Nevertheless, it should be noted that the prevalence in these two studies was based on the use of any complementary therapy after receiving a diagnosis of a disease under study and in the past year, respectively. On the other hand, a stricter criterion of “always use” was used in the present study.

In our study, the complementary therapies with the highest prevalence were fitness walking or strolling, followed by Buddhist prayer or attending temple, and three different types of dietary supplements, including vitamins, calcium, and fish oil. Exercise and dietary supplementation have been reported to be among the most frequently used complementary therapies by Taiwanese patients with breast cancer [23]. Physical inactivity, common in SLE, could contribute to the risk of cardiovascular diseases and comorbid chronic fatigue. A meta-analysis of 11 studies with 469 participants revealed that exercise-based interventions could improve fatigue, depression, and physical fitness in patients with SLE [26].

Prayer is an important spiritual practice that may provide beneficial effects such as emotional healing, reassurance, and hope [27]. Spirituality may improve one’s ability to cope with stressors associated with incurable, chronic, and disabling diseases [28]. A national survey on 28,625 adult Americans found that those with diabetes were significantly more likely to use prayer, which was defined as whether they had ever prayed specifically for their own health [29]. Another recent study on patients with multiple sclerosis also showed that prayer was the most frequently used complementary therapy modality [30]. In Taiwan, a cross-sectional study of female patients with breast cancer receiving anticancer therapy reported a prevalence of 46.4% in the use of spiritual healing, especially prayer, since receiving a diagnosis of breast cancer [22]. Although this prevalence was higher than the 36.8% observed in our study, the findings from both studies reflect that prayer and worship are common practices in local Taiwanese culture. Nevertheless, whether prayer should be considered a complementary therapy, particularly in public health surveillance, remains a matter of debate with broad implications [31, 32]. Future studies should address how best to distinguish prayer from spiritual healing practices under the definition of complementary therapy.

The popular use of dietary supplements by the patients in our study was unsurprising. Vitamin D might reduce SLE disease activity, but evidence from prospective studies is still scarce [33, 34]. Vitamin C intake was found to be inversely associated with the risk of active disease in a cohort study of 279 Japanese female patients with SLE [35]. Similarly, the total serum calcium level has been reported to be inversely associated with SLE disease activity [36]. A meta-analysis showed that bone mineral density was significantly lower in patients with SLE than in healthy controls [37]. In addition, vitamin D deficiency was significantly associated with a higher total/high-density lipoprotein cholesterol ratio as well as with greater disease activity in a cross-sectional study of 290 patients with SLE [38]. Therefore, the reasons for using vitamin supplements by patients with SLE might not be simply because of the possible positive effects on SLE disease activity but also because of their benefits on cardiovascular and bone health.

Fish oil, a rich source of omega-3 polyunsaturated fatty acids, has been shown to offer protective effects on cardiovascular mortality and morbidity in the general population [39]. As SLE is associated with an increased risk of stroke and myocardial infarction [40], patients may consume fish oil to reduce the risk of these serious cardiovascular events. Moreover, a recent meta-analysis of five randomized controlled trials suggested that omega-3 fatty acids could reduce SLE disease activity [41]. A placebo-controlled randomized clinical trial on 32 patients with SLE also showed improvement in the quality of life, disease activity, and biomarkers of inflammation with fish oil supplementation [42].

The results from the multiple logistic regression analysis indicated that age ≥ 40 years, nonoverweight or nonobesity, engagement in vigorous exercise in the past year, a lower SLEDAI-2 K score, and a lower score in the physical health domain of the LupusQoL were significant and independent factors associated with the use of complementary therapies in patients with SLE. Previous research has indicated that complementary therapy use differs by age. Generally, a curvilinear relationship in which middle-aged individuals show the highest use was observed [43]. A comparison study of two national surveys on the use of complementary therapies in Taiwan showed that 40–49 years was the age category with the highest use of complementary therapy [24]. The remaining two factors appeared to indicate that a healthy lifestyle was adopted by the patients. A study based on a nationally representative sample of 23,393 adult Americans indicated that those engaging in multiple healthy behaviours were significantly more likely to use complementary therapies for wellness alone or for a combination of wellness and treatment [44]. Patients with SLE might also use complementary therapies as much to improve their overall wellness as to alleviate disease symptoms and the side effects of treatment. A healthy lifestyle together with the use of complementary therapies might contribute to lower disease activity, as shown by a significantly lower SLEDAI-2 K score. On the other hand, the use of complementary therapies was also associated with a lower score in the physical health domain of the LupusQoL, suggesting that the health-related QoL of the patients was affected by their physical health. A study on patients with chronic rheumatic diseases, including rheumatoid arthritis, SLE, fibromyalgia, and knee osteoarthritis, revealed that complementary therapy use was associated with lower scores in the physical function and bodily pain domains of QoL [45].

Regarding the results of the multiple logistic regression analyses for the five specific types of complementary therapies, the significant factors were found to be dissimilar. Only one factor, engagement in vigorous exercise in the past year, was consistently associated with three different types of complementary therapies. Two factors, including age ≥ 40 years and a higher educational level, were significantly associated with two different types of complementary therapies. The remaining 11 factors were significantly associated with various types of complementary therapies. Of these 14 factors, middle adulthood and higher educational level have consistently been observed in previous studies of complementary therapies among patients with chronic diseases [29, 46, 47]. As mentioned above, a few factors might be related to a healthy lifestyle, including no alcohol use in the past year, no smoking in the past year, engagement in vigorous exercise in the past year, and a sleep duration of 8 hours or more per day. These findings are consistent with the notion that users of complementary therapies tend to take on a proactive approach in maintaining their health [48].

Furthermore, this study found that unemployment was an independent correlate of fitness walking or strolling. Conversely, in the general population, employment was associated with the use of complementary therapies [25]. Nevertheless, prior research has shown that unemployment was associated with an increase in physical activity, possibly by reducing the perceived barriers, such as lack of time, for participation [49]. This may explain the association between unemployment and fitness walking or strolling in our study.

In our patients, a longer disease duration and a poorer self-reported health status were found to be independently associated with the use of vitamins. This finding is consistent with previous studies showing that the use of complementary therapies was correlated with a poorer health status and a longer disease duration in patients with various chronic diseases [50–52]. Approximately one-third of our patients used vitamin supplements on a regular basis. While there is some evidence supporting the association between vitamin D and SLE disease activity, the optimal dosage for supplementation will still need to be established with large-scale studies. In addition, rheumatologists should encourage open communication with their patients regarding the use of supplements to avoid unfavourable interactions with disease treatment [53].

This study also adds to the literature by exploring various domains of health-related QoL between users and nonusers of complementary therapies among patients with SLE. We found that a lower score in the physical health domain but a higher score in the fatigue domain of the LupusQoL were associated with fitness walking or strolling. A possible reason for the latter observation is that only patients who were less affected by fatigue were able to adopt fitness walking or strolling as their regular health maintenance activity. The use of Buddhist prayer or attending temple was associated with a poorer burden on other domains of the LupusQoL. Prayer is often used to deal with negative life issues that do not have other apparent remedies. A review of 16 studies with a total of 1545 study participants concluded that patients with chronic diseases did not pray merely for relief from their physical and mental suffering but rather as a resource to transform their illness experience in a meaningful and positive manner [54]. In addition, the use of calcium supplementation was significantly associated with a lower score in the intimate relationships domain of the LupusQoL. The reason for this association is not clear. Nevertheless, given the significantly increased risk of secondary osteoporosis in patients with SLE [55] and the negative impact of osteoporotic pain on interpersonal relationships [56], this topic warrants further investigation.

There are strengths and limitations to our study. The main strengths of the study included its large sample size and coverage of questions on disease activity and health-related QoL. The limitations of this study included its reliance on self-reported data as ascertained by questionnaires. Moreover, the cross-sectional nature of the study precluded the generation of causal inferences. In addition, our sample was drawn from a regional teaching hospital in southern Taiwan, therefore limiting the generalizability of our findings to other settings.

## Conclusions

Findings from this study in patients with SLE showed that the prevalence of the regular use of any type of complementary therapy was 85.5%. The most popular type of complementary therapy used was fitness walking or strolling, followed by Buddhist prayer or attending temple, vitamin consumption, calcium supplementation, and fish oil supplementation. Multiple logistic regression analyses revealed that different factors were significantly associated with the regular use of any type of complementary therapy and with the five most commonly used types of complementary therapies. Rheumatologists should routinely ask patients about their use of supplements to minimize the risk of interaction with medical therapy.

## Supplementary Information

**Additional file 1.** Survey questions on the use of complementary therapies.

## Data Availability

The datasets generated during and/or analyzed during the current study are available from the corresponding author on reasonable request.

## Abbreviations

ACR: American College of Rheumatology
CI: Confidence interval
OR: Odds ratio
QoL: Quality of life
SD: Standard deviation
SLE: Systemic lupus erythematosus
SLE-DAS: Systemic Lupus Erythematosus Disease Activity Score
SLEDAI-2 K: Systemic Lupus Erythematosus Disease Activity Index 2000
SLICC: Systemic Lupus International Collaborating Clinics Classification Criteria
TCM: Traditional Chinese medicine
