# Supplementary material for: Factors associated with the use of complementary therapies in Taiwanese patients with systemic lupus erythematosus: a cross-sectional study
Source: BMC Complement Med Ther. 2021 Oct 1;21:247. doi: 10.1186/s12906-021-03416-w (PMC8485480; doi:10.1186/s12906-021-03416-w)
Supplement: Supplementary file 1 — Additional file 1. Survey questions on the use of complementary therapies. [file 12906_2021_3416_MOESM1_ESM.docx]

**Additional file 1: Survey questions on the use of complementary therapies**

Factors associated with the use of complementary therapies in Taiwanese patients with systemic lupus erythematosus: a cross-sectional study

1. Body-based and energy therapy

| Type | Use of complementary therapies | | | |
| --- | --- | --- | --- | --- |
|  | Always | Occasionally | Have tried in the past | Never |
| Shiatsu or Tui Na | □ | □ | □ | □ |
| Chiropractic or osteopathic manipulation | □ | □ | □ | □ |
| Gua Sha therapy or cupping | □ | □ | □ | □ |
| Osteopathic manipulation | □ | □ | □ | □ |
| Acupuncture or moxibustion | □ | □ | □ | □ |
| Far-infrared therapy | □ | □ | □ | □ |
| Other:________________ | □ | □ | □ | □ |

2. Mind-body therapy

| Type | Use of complementary therapies | | | |
| --- | --- | --- | --- | --- |
|  | Always | Occasionally | Have tried in the past | Never |
| Qigong or Tai Chi | □ | □ | □ | □ |
| Meditation or spiritual formation | □ | □ | □ | □ |
| Relaxation therapy | □ | □ | □ | □ |
| Aromatherapy | □ | □ | □ | □ |
| Other:________________ | □ | □ | □ | □ |

3. Folk remedies and religious practices

| Type | Use of complementary therapies | | | |
| --- | --- | --- | --- | --- |
|  | Always | Occasionally | Have tried in the past | Never |
| Divination or nameology or fortune-changing | □ | □ | □ | □ |
| Exorcise | □ | □ | □ | □ |
| Buddhist prayer or attending temple | □ | □ | □ | □ |
| Praying or go to church | □ | □ | □ | □ |
| Other:________________ | □ | □ | □ | □ |

4. Exercise therapy

| Type | Use of complementary therapies | | | |
| --- | --- | --- | --- | --- |
|  | Always | Occasionally | Have tried in the past | Never |
| Dancing | □ | □ | □ | □ |
| Fitness workout | □ | □ | □ | □ |
| Jogging | □ | □ | □ | □ |
| Fitness walking or strolling | □ | □ | □ | □ |
| Swimming | □ | □ | □ | □ |
| Cycling | □ | □ | □ | □ |
| Other:________________ | □ | □ | □ | □ |

5. Chinese medicine

| Type | Use of complementary therapies | | | |
| --- | --- | --- | --- | --- |
|  | Always | Occasionally | Have tried in the past | Never |
| Visit Chinese medicine department in hospitals | □ | □ | □ | □ |
| Visit Chinese medicine clinics | □ | □ | □ | □ |
| Visit Chinese herbal shops | □ | □ | □ | □ |
| Self-prescribed herbal remedies | □ | □ | □ | □ |
| Other:________________ | □ | □ | □ | □ |

6. Nutrition supplements

| Type | Use of complementary therapies | | | |
| --- | --- | --- | --- | --- |
|  | Always | Occasionally | Have tried in the past | Never |
| Vitamins ______________ | □ | □ | □ | □ |
| Fish oil | □ | □ | □ | □ |
| Ginkgo | □ | □ | □ | □ |
| Calcium supplement | □ | □ | □ | □ |
| Glucosamine | □ | □ | □ | □ |
| Turmeric | □ | □ | □ | □ |
| Probiotics | □ | □ | □ | □ |
| Other:________________ | □ | □ | □ | □ |

7. Diet therapy

| Type | Use of complementary therapies | | | |
| --- | --- | --- | --- | --- |
|  | Always | Occasionally | Have tried in the past | Never |
| Raw food diet | □ | □ | □ | □ |
| Organic diet | □ | □ | □ | □ |
| Mediterranean diet | □ | □ | □ | □ |
| Low-carbohydrate diet | □ | □ | □ | □ |
| Ketogenic diet | □ | □ | □ | □ |
| Other:________________ | □ | □ | □ | □ |
